# Bisphenol A and Peripheral Arterial Disease: Results from the NHANES

**DOI:** 10.1289/ehp.1104114

**Published:** 2012-05-29

**Authors:** Anoop Shankar, Srinivas Teppala, Charumathi Sabanayagam

**Affiliations:** 1 Department of Epidemiology, West Virginia University School of Public Health, Morgantown, West Virginia, USA; 2 Singapore Eye Research Institute, Singapore; 3 Department of Clinical Sciences, Duke–National University of Singapore Graduate Medical School, Singapore

**Keywords:** ankle–brachial index, bisphenol A, CVD, NHANES, peripheral arterial disease

## Abstract

Background: Bisphenol A (BPA) is a common chemical used in the manufacture of polycarbonate plastics and epoxy resins, and > 93% of U.S. adults have detectable levels of urinary BPA. Recent animal studies have suggested that BPA exposure may have a role in several mechanisms involved in the development of cardiovascular disease (CVD), including weight gain, insulin resistance, thyroid dysfunction, endothelial dysfunction, and oxidative stress. However, few human studies have examined the association between markers of BPA exposure and CVD. Peripheral arterial disease (PAD) is a subclinical measure of atherosclerotic vascular disease and a strong independent risk factor for CVD and mortality.

Objective: We examined the association between urinary BPA levels and PAD in a nationally representative sample of U.S. adults.

Methods: We analyzed data from 745 participants in the National Health and Nutritional Examination Survey 2003–2004. We estimated associations between urinary BPA levels (in tertiles) and PAD (ankle–brachial index < 0.9, *n* = 63) using logistic regression models adjusted for potential confounders (age, sex, race/ethnicity, education, smoking, body mass index, diabetes mellitus, hypertension, urinary creatinine, estimated glomerular filtration rate, and serum cholesterol levels).

Results: We observed a significant, positive association between increasing levels of urinary BPA and PAD before and after adjusting for confounders. The multivariable-adjusted odds ratio for PAD associated with the highest versus lowest tertile of urinary BPA was 2.69 (95% confidence interval: 1.02, 7.09; *p*-trend = 0.01).

Conclusions: Urinary BPA levels were significantly associated with PAD, independent of traditional CVD risk factors.

Bisphenol A (BPA) is a chemical produced in very high volume, with > 2 million metric tons produced worldwide in 2003 (Vandenberg et al. 2009) and used extensively in the manufacture of epoxy resins, polycarbonate plastics, and food and beverage containers (Vandenberg et al. 2009). Consequently, it is one of the most common environmental chemical exposures in humans (Calafat et al. 2005). Data from the nationally representative National Health and Nutrition Examination Survey (NHANES) suggest that detectable levels of BPA are present in the urine of a majority of U.S. adults (Calafat et al. 2005, 2008).

BPA is considered to be an endocrine-disrupting chemical, and it has been shown to have estrogenic and thyroid hormone–disrupting effects in experimental studies (Moriyama et al. 2002; Vandenberg et al. 2009). Recent evidence, especially from animal studies, suggests that BPA exposure may be related to insulin resistance and have a role in weight gain and obesity and the subsequent development of diabetes mellitus (Newbold et al. 2009; Rubin and Soto 2009). Two recent human studies reported that higher urinary BPA levels were associated with self-reported cardiovascular disease (CVD) (Lang et al. 2008; Melzer et al. 2010). However, studies examining the relation between BPA exposure and more objective measures of CVD are needed to support this putative association.

Peripheral arterial disease (PAD) is a subclinical measure of atherosclerotic vascular disease (Newman et al. 1993) that is an independent predictor of subsequent incident CVD (Murabito et al. 2003; Newman et al. 1993). Criqui et al. (1992) reported that among persons initially free of CVD, an ankle–brachial index (ABI) of < 0.9 was associated with a hazard ratio of 6.6 for future risk of death from coronary heart disease even after adjusting for cardiovascular risk factors including age, sex, body mass index (BMI), smoking, and high cholesterol levels. Published guidelines (American Diabetes Association 2003; Hirsch et al. 2006) exist for defining PAD based on the ABI, which can be measured even in large population-based studies (Selvin and Erlinger 2004). In this context, we examined the independent association between urinary BPA levels and PAD among participants in the 2003–2004 NHANES, a representative, multiethnic sample of U.S. adults.

## Methods

The present study is based on data from NHANES 2003–2004. Detailed descriptions of the NHANES study design and methods are available elsewhere [National Center for Health Statistics (NCHS) 2010b]. Briefly, the NHANES survey included a stratified multistage probability sample representative of the civilian noninstitutionalized U.S. population. Selection was based on counties, blocks, households, and individuals within households and included the oversampling of non-Hispanic blacks and Mexican Americans in order to provide stable estimates of these groups. Participants provided written informed consent before their participation, and approval was obtained from the Human Subjects Committee in the U.S. Department of Health and Human Services. Measurements of ABI, a subclinical measure of atherosclerosis (Criqui et al. 1992; Newman et al. 1993), were obtained for the subsample of subjects ≥ 40 years of age.

The present study sample consisted of participants > 40 years of age among whom urinary BPA was available (*n* = 971). We further excluded participants with an ABI ≥ 1.5 (*n* = 3), which is usually related to noncompressible blood vessels in the legs (Newman et al. 1993), as well as those with missing data on covariates included in the multivariable model including level of education, smoking status, serum or fasting glucose levels, systolic or diastolic blood pressure (SBP and DBP, respectively), and cholesterol levels (*n* = 215). This resulted in 753 participants (47.9% women), 63 of whom had PAD. Subjects who were excluded because of missing covariates were in general similar to the general NHANES cohort in terms of age, sex, race/ethnicity, and education (data not shown).

*Exposure measurements.* Age, sex, race/ethnicity, smoking status, alcohol intake (grams per day), level of education, history of diabetes and oral hypoglycemic intake or insulin administration were assessed using a questionnaire (NCHS 2010b). Participants who had not smoked > 100 cigarettes in their lifetimes were considered never smokers; those who had smoked > 100 cigarettes in their lifetimes were considered former smokers if they answered negatively to the question “Do you smoke now?” and current smokers if they answered affirmatively.

Rigorous procedures with quality control checks were used in blood collection, and details about these procedures are provided in the NHANES laboratory/medical technologists procedures manual (NCHS 2010d). We used the modified hexokinase method to measure serum glucose levels at the University of Missouri Diabetes Diagnostic Laboratory (Columbia, MO, USA). Diabetes mellitus was defined, based on the recent guidelines of the American Diabetes Association (2011), as a serum glucose level of > 126 mg/dL after fasting ≥ 8 hr or of > 200 mg/dL for those who fasted < 8 hr before their NHANES visit, a glycosylated hemoglobin value of > 6.5%, or a self-reported current use of oral hypoglycemic medication or insulin. Seated SBP and DBP were measured using a mercury sphygmomanometer according to the American Heart Association and Seventh Joint National Committee recommendations (Chobanian 2003), and up to three measurements were averaged. Patients were considered hypertensive if they reported current use of blood pressure–reducing medication or had SBPs of > 140 mmHg or DBPs of > 90 mmHg (NCHS 2010b).

In NHANES 2003–2004, serum creatinine measurements were conducted at the Collaborative Laboratory Services (Ottumwa, IA, USA) using the Beckman Coulter Synchron LX20 chemistry analyzer (Beckman Coulter, Fullerton, CA, USA) using the Jaffe rate method (kinetic alkaline picrate). Coefficients of variation ranged from 1.5% to 4.3% (NCHS 2010c). In a calibration substudy, serum creatinine assays were performed on 190 stored specimens from NHANES 2003–2004 at the Cleveland Clinic laboratory (Cleveland, OH, USA) using the Roche coupled enzymatic assay that was traceable to gold-standard reference methods, including an isotope dilution mass spectrometric method for serum creatinine using standard references methods [NIST standard reference material (SRM) 967; National Institute of Standards and Technology, Gaithersburg, MD, USA] and confirmed by analysis of CAP LN-24 (College of American Pathologists Creatinine Accuracy Calibration Verification/Linearity Survey) linearity set based on NIST-assigned values (Selvin et al. 2007). There were no significant differences in results between these two measurements, and therefore it was concluded that there was no correction necessary for serum creatinine values in NHANES 2003–2004 (Selvin et al. 2007). The glomerular filtration rate was estimated (eGFR) from serum creatinine using the four-variable Modification of Diet in Renal Disease study equation (Levey et al. 2006) as follows:

eGFR =175 × (serum creatinine in mg/dL)^–1.154^ × (age in years)^–0.203^ × (0.742 if female) × (1.21 if black).

Chronic kidney disease was defined as an eGFR of < 60 mL/min/1.73 m^2^, consistent with definitions of the National Kidney Foundation Kidney Disease Outcome Quality Initiative (KDOQI; National Kidney Foundation 2002) working group and the Kidney Disease Improving Global Outcomes (KDIGO) (Levey et al. 2006).

Previous measures of BPA in biological matrixes involved techniques such as gas chromatography or high performance liquid chromatography (Ye et al. 2005). To achieve enhanced sensitivity and selectivity over previous methods, in the current NHANES, measures of environmental phenols were derivatized to alkyl or acyl derivatives before gas chromatography–mass spectrometry analysis (NCHS 2010a). The lower limit of detection for BPA concentrations was 0.36 ng/mL.

*Main outcome of interest: PAD.* For study subjects with at least one arm and weighing ≤ 400 lb, supine SBP was measured with blood pressure cuffs on the right arm, compressing the brachial artery and the two posterior tibial arteries (NCHS 2010a). For subjects 40–59 years of age, two measurements were taken at each site and averaged, and for patients ≥ 60 years of age, one measure was taken at each site. For subjects with conditions precluding the measurement of the right arm, left brachial artery SBP was taken. Left and right ABI values were calculated as the ratio of left and right ankle SBP, respectively, to arm SBP. The smallest of the left and right ABI measurements was used. Patients with ABIs ≥ 1.5 (*n* = 3) are expected to have severe arterial rigidity and were excluded from the present analyses (Newman et al. 1993). For these analyses, PAD was defined as ABI < 0.9 (American Diabetes Association 2011; Selvin and Erlinger 2004). Because of the selection criteria for ABI measurements in NHANES (subjects ≥ 40 years of age), subjects who had ABI measurements taken were significantly older than the general NHANES cohort but were otherwise similar with respect to demographic factors (data not shown).

*Statistical analysis.* Urinary BPA was categorized into tertiles (< 1.4 ng/mL, 1.4–3.5 ng/mL, > 3.5 ng/mL). We hypothesized that high BPA levels are associated with PAD. Odds ratios (ORs) and 95% confidence intervals (CIs) for PAD in association with BPA were calculated by taking the lowest tertile (tertile 1) as the referent, using multivariable logistic regression models. We used two models: one adjusted only for age and sex, and a multivariable model that also adjusted for race/ethnicity (non-Hispanic whites, non-Hispanic blacks, Mexican Americans, others), education (< high school, high school, > high school), household income (< $25,000, $25,000–54,999, ≥ $55,000), smoking status (never, former, current), pack-years of smoking, alcohol intake (nondrinker, moderate drinker, heavy drinker), BMI (normal, overweight, obese), hypertension (present, absent), diabetes (present, absent), urinary creatinine (milligrams per deciliter), eGFR (milliliters per minute per 1.73 m^2^), and total cholesterol (milligrams per deciliter). Trends in the OR of PAD across increasing urinary BPA categories were determined by modeling BPA as an ordinal variable. We examined effect modification by performing stratified analysis by categories of sex, race/ethnicity, BMI, diabetes mellitus, and hypertension. We also conducted formal statistical tests for interaction by including multiplicative cross-product interaction terms in the multivariable logistic regression models; *p* < 0.1 was interpreted as a statistically significant interaction. Sample weights that account for the unequal probabilities of selection, oversampling, and non-response were applied for all analyses using SAS (version 9.2; SAS Institute Inc., Cary, NC, USA) and SUDAAN software (version 10; RTI International, Research Triangle Park, NC, USA); SEs were estimated using the Taylor series linearization method.

## Results

The median (interquartile range) of urinary BPA levels was 2.30 (3.60) ng/mL; the 25th, 50th, and 75th percentile values were 1.00, 2.30, and 4.60 ng/mL, respectively. Participants with higher levels of BPA were more likely to be men, non-Hispanic blacks, and former or current smokers and had higher levels of urinary creatinine. Table 1 shows these characteristics for the study population by teriles of urinary BPA levels. Overall, we observed a positive association between increasing BPA levels and PAD on the basis of the age- and sex-adjusted model and the multivariable-adjusted model, with significant (*p* < 0.05) trend tests (Table 2). This positive association persisted when BPA was analyzed as a continuous variable (1-SD increase in log-transformed BPA).

**Table 1:** Characteristics of the study population by tertiles of urinary BPA levels.

Characteristic	Tertile 1 (< 1.4 ng/mL)	Tertile 2 (1.4–3.6 ng/mL)	Tertile 3 (> 3.6 ng/mL)
Unweighted sample size		253		240		252
Age (years)		56.7 ± 0.6		55.6 ± 0.7		55.5 ± 0.9
Female (%)		56.5		54.8		42.7
Race/ethnicity (%)						
Non-Hispanic whites		76.3		81.4		75.0
Non-Hispanic blacks		3.7		9.1		14.4
Mexican Americans and others		20.0		9.5		10.6
Education (%)						
< High school		16.5		19.7		16.8
High school		24.0		29.5		27.6
> High school		59.5		50.8		55.6
Income (%)						
< $25,000		20.1		23.3		21.2
$25,000–$54,999		29.9		40.1		40.0
≥ $55,000		50.0		36.6		38.8
Smoking status (%)						
Never		51.7		52.2		39.2
Former		30.8		30.1		37.5
Current		17.5		17.7		23.3
Pack-years of smoking		12.6 ± 1.6		13.5 ± 1.0		17.4 ± 2.2
Alcohol intake (%)						
Nondrinker		29.9		41.1		39.5
Moderate drinker		55.1		42.5		40.0
Heavy drinker		15.0		16.4		20.5
BMI (%)						
Normal weight (< 25 kg/m2)		31.5		32.5		23.3
Overweight (25–30 kg/m2)		38.7		35.0		38.6
Obese (BMI ≥ 30 kg/m2)		29.8		32.5		38.1
Diabetes (%)		11.0		14.4		16.1
Hypertension (%)		43.4		45.9		51.5
Urinary creatinine (mg/dL)		70.2 ± 5.2		120.5 ± 4.3		161.8 ± 5.2
eGFR (mL/min/1.73 m2)		81.5 ± 1.3		80.5 ± 1.3		80.1 ± 1.9
Total cholesterol (mg/dL)		210.9 ± 2.3		209.6 ± 3.5		207.0 ± 3.3
Urinary BPA (ng/mL)		0.7 ± 0.02		2.4 ± 0.05		10.0 ± 1.1
PAD (%)		2.8		4.1		9.1
Data presented are mean ± SE except where indicated.						

**Table 2:** Association between urinary BPA and PAD.

BPA (ng/mL)	Unweighted sample size (weighted PAD prevalence)	Age, sex-adjusted OR (95% CI)	Multivariable-adjusted OR (95% CI)a
Tertile 1 (< 1.4)		253 (2.8%)		1 (referent)		1 (referent)
Tertile 2 (1.4–3.6)		240 (4.1%)		1.53 (0.39, 6.04)		1.10 (0.22, 5.39)
Tertile 3 (> 3.6)		252 (9.1%)		3.73 (2.03, 6.86)		2.69 (1.02, 7.09)
p-Trend				< 0.0001		0.01
1-SD increase in log-transformed BPA (ng/mL)b				1.57 (1.30, 1.90)		1.38 (1.11, 1.72)
aAdjusted for age, sex, race/ethnicity, education, income, smoking status, pack-years of smoking, alcohol intake, BMI, hypertension, diabetes, urinary creatinine, eGFR, and total cholesterol. b1 SD of log-transformed BPA = 1.15 ng/mL.						

In a supplementary analysis, we examined the association between BPA levels and PAD after excluding *n* = 10 subjects with very high BPA values (levels > 30 ng/mL). The results were found to be essentially similar. The multivariable OR of PAD associated with 1 SD of log-transformed BPA was 1.60 (1.16–2.21). We also examined the association between increasing tertiles of BPA and self-reported CVD, and the magnitude of association was found to be weaker than that for PAD. Compared to tertile 1 of BPA (referent), the multivariable-adjusted OR (95% CI) of self-reported CVD was 1.09 (0.18–6.60) for tertile 2, and 1.83 (1.01–3.31) for tertile 3 (*p*-trend = 0.02).

## Discussion

In a large multiethnic, nationally representative sample, we found that increasing serum BPA levels were strongly associated with PAD. The observed association was independent of the confounding factors of smoking, BMI, alcohol intake, diabetes mellitus, hypertension, and serum cholesterol level. Our study adds to the emerging evidence suggesting a role for environmental exposure to BPA in cardiovascular disease in humans (Lang et al. 2008; Melzer et al. 2010). Furthermore, because PAD and low ABI are markers of atherosclerosis, our findings suggest that potential effects of BPA on atherosclerosis may be a mechanism underlying the previously reported association between BPA exposure and self-reported CVD (Lang et al. 2008; Melzer et al. 2010).

BPA is an environmental chemical used as a constituent monomer in polycarbonate plastics, which are used extensively in drink containers and food packaging and in the production of oxidants used in the lining of canned goods (Vandenberg et al. 2009). Exposure to BPA is believed to be mainly through dietary intake with additional exposure through water, dental sealants, inhalation of household dusts, and exposure through skin (Vandenberg et al. 2009). Recent studies from NHANES data have documented that > 93–95% of the general U.S. population has measurable concentrations of BPA metabolites in their urine (Calafat et al. 2005, 2008).

Several lines of recent evidence suggest that an association between urinary BPA levels and PAD may be biologically plausible. Animal studies have suggested that BPA exposure may have a role in CVD development through several mechanisms, including the role of BPA in weight gain and obesity development potentially through its action on preadipocytes (Masuno et al. 2005; Phrakonkham et al. 2008), role as an estrogen (Rubin and Soto 2009), potential interactions with estrogen-related receptor gamma (Matsushima et al. 2008), actions as a thyroid hormone antagonist (Moriyama et al. 2002), role as a peroxisome proliferator–activated receptor gamma antagonist (Wright et al. 2000) and its role in influencing pancreatic endocrine function (Ropero et al. 2008). Alonso-Magdalena et al. (2006) showed that mice with long-term exposure to environmentally relevant levels of BPA developed hyperinsulinemia, insulin resistance, and glucose intolerance. Furthermore, in animal models BPA has been shown to induce endothelial cell injury mediated through oxidative stress (Hennig et al. 2002; Ooe et al. 2005; Stegeman et al. 1995) and elevations in lipids (Marmugi et al. 2012). However, there are few studies in humans for comparison.

Two of the few human studies (Lang et al. 2008; Melzer et al. 2010) reported positive associations between urinary BPA levels and self-reported CVD. Also, BPA levels were found to be associated with abnormal liver function enzymes and higher levels of fasting glucose, insulin, and HOMA-IR (homeostasis model of assessment—insulin resistance) (Lang et al. 2008; Melzer et al. 2010), all points suggesting that BPA may have a role in CVD. It is in this context that our results on objectively measured ABI to define PAD are important. In the present study, we have demonstrated a significant, positive association between higher BPA levels and PAD that persisted after adjustment for multiple confounders.

The main strengths of our study include its nationally representative sample and use of rigorous study methods to collect the data and the availability of extensive data on confounders (NCHS 2010a, 2010b). The main study limitation is that the present study is cross-sectional in nature, therefore making it impossible to confirm that exposure preceded the outcome. Another limitation is the possibility of residual confounding. For example, despite adjusting for education and income, the observed association between BPA levels and PAD may be at least partly explained by residual confounding by low socioeconomic status. Future prospective studies are required to confirm or disprove our findings. A third limitation is that because ABI measures were available only in NHANES 2003–2004, we are not able to examine data from the recent NHANES cycles such as NHANES 2005–2006, where BPA levels may be lower. Fourth, the use of urinary BPA levels measured from a single spot sample is likely to result in some misclassification because of the large variability of BPA. Therefore, to provide the best approach for BPA exposure assessment, expert panels have recommended collection of multiple (rather than single) spot urine samples and study designs that consider important exposure contributors such as the time of the day of sampling (e.g., in relation to food consumption) and the time of last urination (World Health Organization/Food and Agriculture Organization of the United Nations Expert Panel 2011). Finally, urinary BPA levels may not be reflective of the free, unconjugated, circulating BPA in blood, which is considered to be the biologically active form. Future studies examining the health effects of BPA should preferably measure this free, unconjugated part of BPA in serum.

## Conclusions

In a nationally representative sample of U.S. adults, higher BPA levels were positively associated with PAD after adjusting for confounding factors such as age, sex, smoking, BMI, alcohol intake, diabetes, hypertension, and cholesterol levels. Although our findings must be confirmed in other studies, they provide preliminary evidence that environmental exposure to BPA may contribute to PAD.

## References

[r1] Alonso-Magdalena P, Morimoto S, Ripoll C, Fuentes E, Nadal A. (2006). The estrogenic effect of bisphenol A disrupts pancreatic β-cell function *in vivo* and induces insulin resistance.. Environ Health Perspect.

[r2] American Diabetes Association (2003). Peripheral arterial disease in people with diabetes.. Diabetes Care.

[r3] American Diabetes Association (2011). Diagnosis and classification of diabetes mellitus.. Diabetes Care.

[r4] Calafat AM, Kuklenyik Z, Reidy JA, Caudill SP, Ekong J, Needham LL (2005). Urinary concentrations of bisphenol A and 4-nonylphenol in a human reference population.. Environ Health Perspect.

[r5] Calafat AM, Ye X, Wong LY, Reidy JA, Needham LL (2008). Exposure of the U.S. population to bisphenol A and 4-*tertiary*-octylphenol: 2003–2004.. Environ Health Perspect.

[r6] Chobanian AV, Bakris GL, Black HR, Cushman WC, Green LA, Izzo JL (2003). Seventh report of the Joint National Committee on Prevention, Detection, Evaluation, and Treatment of High Blood Pressure.. Hypertension.

[r7] Criqui MH, Langer RD, Fronek A, Feigelson HS, Klauber MR, McCann TJ (1992). Mortality over a period of 10 years in patients with peripheral arterial disease.. N Engl J Med.

[r8] Hennig B, Hammock BD, Slim R, Toborek M, Saraswathi V, Robertson LW (2002). PCB-induced oxidative stress in endothelial cells: modulation by nutrients.. Int J Hyg Environ Health.

[r9] Hirsch AT Haskal ZJ Hertzer NR Bakal CW Creager MA Halperin JL 2006 ACC/AHA 2005 Practice Guidelines for the management of patients with peripheral arterial disease (lower extremity, renal, mesenteric, and abdominal aortic): a collaborative report from the American Association for Vascular Surgery/Society for Vascular Surgery, Society for Cardiovascular Angiography and Interventions, Society for Vascular Medicine and Biology, Society of Interventional Radiology, and the ACC/AHA Task Force on Practice Guidelines (Writing Committee to Develop Guidelines for the Management of Patients With Peripheral Arterial Disease). Circulation 113 e463 e654 doi:10.1161/CIRCULATIONAHA.106.174526 16549646

[r10] Lang IA, Galloway TS, Scarlett A, Henley WE, Depledge M, Wallace RB (2008). Association of urinary bisphenol A concentration with medical disorders and laboratory abnormalities in adults.. JAMA.

[r11] Levey AS, Coresh J, Greene T, Stevens LA, Zhang YL, Hendriksen S (2006). Using standardized serum creatinine values in the modification of diet in renal disease study equation for estimating glomerular filtration rate.. Ann Intern Med.

[r12] Marmugi A, Ducheix S, Lasserre F, Polizzi A, Paris A, Priymenko N (2012). Low doses of bisphenol A induce gene expression related to lipid synthesis and trigger triglyceride accumulation in adult mouse liver.. Hepatology.

[r13] Masuno H, Iwanami J, Kidani T, Sakayama K, Honda K. (2005). Bisphenol A accelerates terminal differentiation of 3T3-L1 cells into adipocytes through the phosphatidylinositol 3-kinase pathway.. Toxicol Sci.

[r14] Matsushima A, Teramoto T, Okada H, Liu X, Tokunaga T, Kakuta Y (2008). ERRγ tethers strongly bisphenol A and 4-α-cumylphenol in an induced-fit manner.. Biochem Biophys Res Commun.

[r15] Melzer D Rice NE Lewis C Henley WE Galloway TS 2010 Association of urinary bisphenol A concentration with heart disease: evidence from NHANES 2003/06. PLoS One 5 e8673 doi:10.1371/journal.pone.0008673 [Online 13 January 2010] 20084273 PMC2800195

[r16] Moriyama K, Tagami T, Akamizu T, Usui T, Saijo M, Kanamoto N (2002). Thyroid hormone action is disrupted by bisphenol A as an antagonist.. J Clin Endocrinol Metab.

[r17] Murabito JM, Evans JC, Larson MG, Nieto K, Levy D, Wilson PW (2003). The ankle–brachial index in the elderly and risk of stroke, coronary disease, and death: the Framingham Study.. Arch Intern Med.

[r18] National Kidney Foundation (2002). K/DOQI Clinical practice guidelines for chronic kidney disease: evaluation, classification, and stratification.. Am J Kidney Dis.

[r19] NCHS (National Center for Health Statistics) (2010a). 2003–2004 National Health and Nutrition Examination Survey: 2003–2004 Data Documentation, Codebook, and Frequencies—Lower Extremity Disease–Ankle Brachial Blood Pressure Index (LEXAB_C).. http://www.cdc.gov/nchs/nhanes/nhanes2003-2004/LEXAB_C.htm.

[r20] NCHS (National Center for Health Statistics) (2010b). 2003–2004 National Health and Nutrition Examination Survey: Survey Operations Manuals, Brochures, Consent Documents.. http://www.cdc.gov/nchs/nhanes/nhanes2003-2004/current_nhanes_03_04.htm.

[r21] NCHS (National Center for Health Statistics) (2010c). Laboratory Procedure Manual for Serum Creatinine.. http://www.cdc.gov/nchs/data/nhanes/nhanes_03_04/l40_c_met_creatinine.pdf.

[r22] NCHS (National Center for Health Statistics) (2010d). NHANES 2003–2004 Data Release: January 2006. General Information about the NHANES 2003–2004 Laboratory Methodology and Public Data Files.. http://www.cdc.gov/nchs/data/nhanes/nhanes_03_04/lab_c_generaldoc.pdf.

[r23] Newbold RR, Padilla-Banks E, Jefferson WN (2009). Environmental estrogens and obesity.. Mol Cell Endocrinol.

[r24] Newman AB, Siscovick DS, Manolio TA, Polak J, Fried LP, Borhani NO, Cardiovascular Heart Study (CHS) Collaborative Research Group (1993). Ankle–arm index as a marker of atherosclerosis in the Cardiovascular Health Study.. Circulation.

[r25] Ooe H, Taira T, Iguchi-Ariga SM, Ariga H (2005). Induction of reactive oxygen species by bisphenol A and abrogation of bisphenol A–induced cell injury by DJ-1.. Toxicol Sci.

[r26] Phrakonkham P, Viengchareun S, Belloir C, Lombes M, Artur Y, Canivenc-Lavier MC (2008). Dietary xenoestrogens differentially impair 3T3-L1 preadipocyte differentiation and persistently affect leptin synthesis.. J Steroid Biochem Mol Biol.

[r27] Ropero AB, Alonso-Magdalena P, Garcia-Garcia E, Ripoll C, Fuentes E, Nadal A (2008). Bisphenol-A disruption of the endocrine pancreas and blood glucose homeostasis.. Int J Androl.

[r28] Rubin BS, Soto AM (2009). Bisphenol A: Perinatal exposure and body weight.. Mol Cell Endocrinol.

[r29] Selvin E, Erlinger TP (2004). Prevalence of and risk factors for peripheral arterial disease in the United States: results from the National Health and Nutrition Examination Survey, 1999–2000.. Circulation.

[r30] Selvin E, Manzi J, Stevens LA, Van LF, Lacher DA, Levey AS (2007). Calibration of serum creatinine in the National Health and Nutrition Examination Surveys (NHANES) 1988–1994, 1999–2004.. Am J Kidney Dis.

[r31] Stegeman JJ, Hahn ME, Weisbrod R, Woodin BR, Joy JS, Najibi S (1995). Induction of cytochrome P4501A1 by aryl hydrocarbon receptor agonists in porcine aorta endothelial cells in culture and cytochrome P4501A1 activity in intact cells.. Mol Pharmacol.

[r32] Vandenberg LN, Maffini MV, Sonnenschein C, Rubin BS, Soto AM (2009). Bisphenol-A and the great divide: a review of controversies in the field of endocrine disruption.. Endocr Rev.

[r33] World Health Organization/Food and Agriculture Organization of the United Nations Expert Panel (2011). Toxicological and Health Aspects of Bisphenol A.. http://whqlibdoc.who.int/publications/2011/97892141564274_eng.pdf.

[r34] Wright HM, Clish CB, Mikami T, Hauser S, Yanagi K, Hiramatsu R (2000). A synthetic antagonist for the peroxisome proliferator–activated receptor γ inhibits adipocyte differentiation.. J Biol Chem.

[r35] Ye X, Kuklenyik Z, Needham LL, Calafat AM (2005). Automated on-line column-switching HPLC-MS/MS method with peak focusing for the determination of nine environmental phenols in urine.. Anal Chem.

